# Point-of-Care Ultrasound Use in Confirmed Acute Aortic Syndrome: A Five-Year Retrospective Cohort Study at the Ljubljana Emergency Medical Unit

**DOI:** 10.3390/jcm15208189

**Published:** 2026-10-21

**Authors:** Pika Brezigar, Jan Kukavica, Borut Jug, Hugon Možina

**Affiliations:** 1 Faculty of Medicine, University of Ljubljana, SI-1000 Ljubljana, Slovenia; pika.brezigar@gmail.com (P.B.); jan.kukavica@gmail.com (J.K.); 2 Department of Vascular Diseases, University Medical Centre Ljubljana, SI-1000 Ljubljana, Slovenia; borut.jug@kclj.si; 3 Emergency Medical Unit, University Medical Centre Ljubljana, SI-1000 Ljubljana, Slovenia

**Keywords:** aortic dissection, point-of-care ultrasound, emergency medicine

## Abstract

**Background/Objectives:** Point-of-care ultrasound (POCUS) is increasingly used in emergency care, but data on its use in acute aortic syndrome (AAS) remains limited. This single-center retrospective cohort described POCUS use among patients with confirmed AAS and explored factors associated with POCUS use and time to completion of diagnostic workup. **Methods:** Records coded ICD10 I71.0–I71.8 from the Emergency Medical Unit, University Medical Centre Ljubljana (2019–2023) were reviewed. Clinical, imaging, and outcome data were extracted, and diagnoses were confirmed by two investigators through manual chart review of ED documentation, radiology reports, and specialist consultation notes. Fisher’s exact and Wilcoxon rank sum tests were used for comparisons. Associations with POCUS use and time to completion of diagnostic workup (defined from arrival at triage to completion of the emergency department workup) were assessed with univariable logistic regression and linear regression models, respectively. Survival was explored using univariable Cox proportional-hazards models. Statistical significance was set at *p* < 0.05. **Results:** Among 120,109 emergency department visits, 73 patients had confirmed AAS (type A: 48; type B: 17; intramural hematoma: 8). POCUS was performed in 62% of patients, more often in those who were hemodynamically unstable (87.5% vs. 54.4%; *p* = 0.020) or had unclear presentations (40% vs. 7%; *p* = 0.003). Median time to completion of diagnostic workup was shorter with POCUS (110 vs. 213 min), though not statistically significant (*p* = 0.069). Hemodynamic instability was associated with higher odds of POCUS use (OR 5.87, 95% CI 1.22–28.2; *p* = 0.028), whereas a clear clinical presentation was associated with lower odds of POCUS use (OR 0.12, 95% CI 0.02–0.55; *p* = 0.007). Lower triage level was associated with longer time to completion of diagnostic workup (time ratio 5.43, 95% CI 2.21–13.4; *p* < 0.001 for urgent vs. immediate triage), whereas very high D-dimer levels were associated with shorter time to completion of diagnostic workup (time ratio 0.44, 95% CI 0.21–0.93; *p* = 0.031 for highest vs. lowest quartile). In-hospital mortality was 14%, with only age associated with mortality (HR 1.06 per year, 95% CI 1.02–1.11; *p* = 0.007). **Conclusions:** In this single-center retrospective AAS cohort, POCUS was used more frequently in unstable and diagnostically unclear patients but was not associated with time to completion of diagnostic workup. Findings are descriptive and exploratory; they do not establish that POCUS accelerates diagnosis or improves outcomes.

## 1. Introduction

Point of care ultrasound (POCUS) has become an important component of contemporary emergency medicine. It provides real-time, bedside imaging that physicians can obtain, interpret, and immediately integrate into clinical decision-making. Unlike formal imaging, POCUS can be performed during the initial assessment, repeated as the patient’s condition evolves, and used to guide both diagnosis and procedures. Its applications extend across resuscitation, undifferentiated shock, acute dyspnea, chest pain, abdominal pain, trauma, vascular disease, and ultrasound-guided vascular access [1,2].

POCUS is particularly valuable when the differential diagnosis is broad and rapid diagnostic clarification is needed before laboratory results or advanced imaging are available. In many emergency situations, including aortic emergencies, POCUS can provide immediate information at the bedside; it offers clinicians a speed advantage for rapid diagnosis, rapid awareness, and rapid intervention [3]. A systematic review found that incorporating POCUS into the assessment of patients with undifferentiated shock increased diagnostic accuracy from approximately 45–60% to 80–89% [4]. In a prospective study of patients presenting with acute dyspnea, the median time to a POCUS-based diagnosis was 16 min, compared with 170 min for the final composite diagnosis, and diagnostic agreement was considered good [5].

In addition, a randomized study of patients presenting with acute abdominal pain found that POCUS reduced the number of preliminary differential diagnoses and was associated with shorter emergency department stay among both discharged and admitted patients [6]. These findings suggest that POCUS may have the potential to reduce diagnostic uncertainty and shorten the time needed to identify high-risk patients, initiate treatment, obtain consultation, and determine disposition.

This broader role of POCUS is relevant to patients with acute aortic syndrome (AAS), in whom the initial clinical presentation is highly variable and overlaps with other emergent causes of chest pain, dyspnea, abdominal pain, syncope, and shock. AAS comprises life-threatening conditions characterized by disruption of aortic wall integrity, most commonly aortic dissection (AD), intramural hematoma (IMH), and penetrating atherosclerotic ulcer (PAU), all of which carry a risk of rupture [7]. Mortality in untreated patients with dissections involving the ascending aorta (Stanford type A) rises by 2.6% per hour, making timely diagnosis essential [8]. Emergency physicians fail to recognize AD in up to 38% of cases, and among those correctly identified, one quarter receive the diagnosis more than 24 h after presentation [9,10]. Autopsy data spanning nearly six decades similarly show that 63% of AD cases were diagnosed only postmortem, with little improvement over time [11].

Sudden, severe chest pain radiating to the back, neck, or shoulders occurs in over 90% of cases. The pain is most often described as sharp; a tearing quality is considered more characteristic despite being less frequently reported [12,13,14,15]. The presentation is highly variable and may include abdominal pain, dyspnea, syncope, pulse deficits, limb ischemia, or neurological symptoms. Because these findings are frequently nonspecific or atypical, and pain may even be absent, history and physical examination alone are frequently insufficient for establishing a definitive diagnosis [12,13,14,15,16].

Contrast-enhanced computed tomography angiography (CTA) remains the diagnostic gold standard for AD, with a sensitivity of about 95% and specificity approaching 100%, and is recommended as the primary imaging modality in current American and European guidelines [7,17,18,19]. However, POCUS is increasingly used as an initial bedside assessment, especially in hemodynamically unstable patients in whom transport to CT is risky and logistically challenging [20]. POCUS offers a focused cardiac, lung, and aortic examination that relies on the physician’s understanding of the expected pathology and its sonographic features, enabling quick identification of life threatening conditions. It may reveal alternative diagnoses while also detecting direct or indirect signs of AD, including an intimal flap, aortic root dilation, aortic regurgitation, pericardial effusion, or cardiac tamponade [21,22,23]. In this context, POCUS should be viewed as an adjunct to clinical assessment and definitive imaging rather than a replacement. Its potential value lies in providing immediate diagnostic information at the bedside during time-sensitive assessment and procedures, thereby supporting earlier clinical decisions without transporting an unstable patient [3].

Evidence on POCUS use in larger cohorts of patients with AAS remains limited, and most publications are case reports. The first study evaluating POCUS in a substantial AD cohort was published in 2012, reporting its use in 52% of patients without prolonging overall evaluation time [24]. A systematic review of emergency physician-performed ultrasound of the thoracic aorta found that POCUS is highly specific but insufficiently sensitive to rule out AD, with sensitivities of 41–91% and specificities of 94–100% when an intimal flap was visualized [25]. A multicenter prospective study has since evaluated a standardized POCUS protocol combining transthoracic echocardiography and abdominal aortic ultrasound for the emergent evaluation of AD [26]. However, the patient and clinical factors associated with POCUS use, and whether its use is associated with a shorter time to completion of diagnostic workup in confirmed cases, have not been systematically examined. This study was not designed to assess the diagnostic accuracy of POCUS. Rather, among patients with a confirmed diagnosis of AAS, we evaluated the utilization of POCUS and factors associated with its use and compared time to completion of diagnostic workup between patients evaluated with and without POCUS.

## 2. Materials and Methods

### 2.1. Study Design and Population

**Figure 1:**
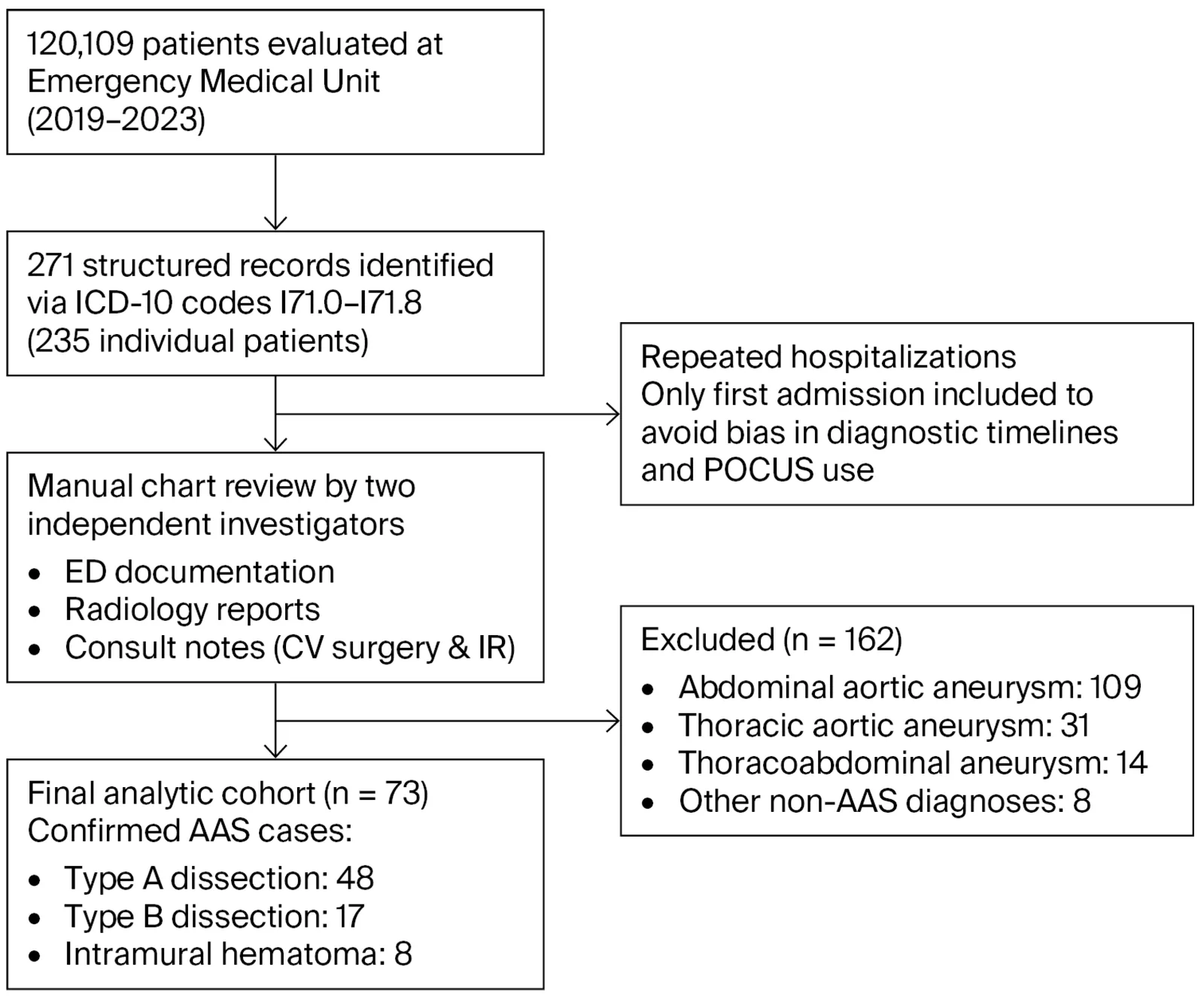
Flow of participants through the study.

We conducted a retrospective cohort analysis of patients with confirmed AAS at the Emergency Medical Unit of the University Medical Centre Ljubljana, Slovenia between 1 January 2019 and 31 December 2023. During this period, 120,109 patients were examined in the department. To identify potential AAS cases, we screened all records coded under ICD10 diagnoses I71.0–I71.8. This search yielded 271 structured medical records corresponding to 235 individual patients. After detailed review of clinical documentation and imaging reports, 73 patients met the criteria for confirmed AAS: type A AD (n = 48), type B AD (n = 17), or intramural hematoma (IMH; n = 8) (Figure 1). All AAS diagnoses and subtypes (type A AD, type B AD, and intramural hematoma) were classified by a multidisciplinary vascular team comprising a cardiovascular surgeon, a radiologist, and a cardiologist at the time of the patient’s clinical evaluation.

The review included all available emergency department documentation, radiology reports, and specialist consultation notes from cardiovascular surgery and interventional radiology to ensure accurate case ascertainment.

The most common excluded diagnoses were abdominal aortic aneurysm (n = 109), thoracic aortic aneurysm (n = 31), and thoracoabdominal aortic aneurysm (n = 14). For patients who were hospitalized more than once for AD or IMH during the study period, only the first hospitalization was included to avoid bias related to prior disease history influencing diagnostic timelines or POCUS use.

### 2.2. Data Collection

We recorded demographic information (age at presentation, sex), time intervals in the diagnostic pathway (time to first evaluation, time to final diagnosis), past medical history and risk factors (arterial hypertension, Marfan syndrome), clinical assessment at admission (triage category, hemodynamic status, clarity of clinical presentation), AAS subtype (type A AD, type B AD, IMH), performance of POCUS and its ultrasound findings (dissection membrane, cardiac tamponade, aortic regurgitation, intimal flap in the descending aorta), performance of CTA, laboratory results (Ddimer, troponin), interventions in the emergency department (intravenous βblockers, vasodilators), subsequent treatment, and final outcomes (surgical or endovascular management, in-hospital survival, date of death).

Triage category was assigned using the Manchester Triage System (red = immediate, orange = very urgent, yellow = urgent). Depending on the point of first medical contact, triage was performed by triage nurses in the emergency department admission office, emergency physicians, or paramedics in the prehospital environment.

POCUS examinations were performed by attending physicians in the Emergency Medical Unit. All attending physicians are specialists in either general internal medicine or emergency medicine and have received formal POCUS training as part of their specialist training prior to assuming a position in the Emergency Medical Unit. The examination was not performed according to a single mandatory protocol; the views and target organs were selected according to the treating physician’s clinical assessment. It should be noted that some of these ultrasound signs, particularly aortic regurgitation and cardiac tamponade, are not specific for AAS and may be observed in other cardiovascular conditions. The presence of an intimal flap or dissection membrane was recorded strictly as documented in the medical record, based on the discretion and interpretation of the physician performing the ultrasound examination. Cardiac tamponade was recorded only when echocardiographic features of hemodynamic compromise were documented, including visible diastolic right ventricular inflow collapse or equivalent signs of tamponade physiology. Intimal flap in the descending aorta was recorded only when the medical documentation explicitly described a visible intimal flap or dissection membrane in the descending aorta.

In all patients classified as having undergone POCUS, the examination was performed during the initial emergency-unit evaluation before definitive diagnosis. Because of the retrospective design, exact timing relative to CTA and systematic archiving or independent verification of POCUS images and findings were not consistently available. Therefore, POCUS findings were analyzed as documented bedside findings rather than validated diagnostic-test results.

Based on history and clinical status, cases were classified as having a clear clinical presentation using the AORTAs simplified score (AORTAs). It stratifies patients into low- (≤1 point) or high- (≥2 points) risk for AD and automatically categorizes hemodynamically unstable patients as high-risk. For the purpose of this study, an AORTAs score ≥2 (high-risk) was classified as a clear clinical presentation, whereas a score ≤1 (low-risk) was classified as an unclear clinical presentation. Two independent investigators calculated the AORTAs score for every patient by reviewing the initial emergency-unit documentation; discrepancies were resolved through discussion until consensus was reached for all cases. This scoring system was selected due to its advantages over the Aortic Dissection Detection Risk Score (ADDRS), offering a simpler and faster assessment based on six key features. In the study by Morello et al., AORTAs demonstrated higher sensitivity, greater overall diagnostic accuracy, and more identified AAS cases, with only minimal loss of specificity. Its design is another strength: it was developed within a prospective multicenter study and has been validated in two independent cohorts, whereas ADDRS is based on retrospective database analysis and has been less consistently validated [27].

Since there is no universally accepted definition, hemodynamic instability was defined as a heart rate >100 beats per minute or systolic blood pressure <90 mmHg. This is consistent with existing research, in which tachycardia and hypotension are the most consistently recognized and clinically useful indicators of compromised circulation [28].

Time to first evaluation and time to completion of diagnostic workup was derived from structured timestamps recorded in the emergency department documentation and discharge summaries. For each patient, four time points were available: arrival, first physician examination, completion of emergency department workup, and transfer/discharge. Time zero was defined as the time of arrival at triage. Time to first evaluation was calculated as the interval between arrival and the timestamp for first physician contact. The principal time outcome was calculated from arrival to the timestamp for completion of emergency department workup, corresponding to the time when the patient was considered ready for transfer or discharge from the emergency department (i.e., the duration of the emergency department diagnostic pathway). The median time to examination of 0 min reflects the timestamp resolution in our system, which records times in whole minutes; in many cases, the first physician contact occurred within the same minute as triage registration, resulting in a calculated interval of 0 min. In the majority of patients, final diagnosis was established by CTA prior to completion of the emergency department workup. In two patients who died in the emergency department before CTA could be performed, the time of death was used as the timestamp for both completions of workup and transfer/discharge; diagnoses in both cases were subsequently confirmed by autopsy reports. For the remaining patient who did not undergo CTA, final diagnosis was based on alternative definitive imaging or specialist confirmation, and the corresponding workup completion timestamp was used.

### 2.3. Statistical Analysis

Statistical analyses were performed using R (version 4.2.3; R Foundation for Statistical Computing, Vienna, Austria). Because this retrospective study included all eligible AAS patients identified within the study period, no prior sample-size calculation was performed; the available sample size was determined by case ascertainment.

Continuous variables were presented as median with interquartile range; dichotomous variables were presented as number and percentage. Between-group continuous variables were compared using the Wilcoxon rank sum test; dichotomous variables were compared using Fisher’s exact test. The primary regression analyses were univariable. Predictors of POCUS use were assessed using separate logistic regression models for age, sex, triage category, hemodynamic compromise, clarity of clinical presentation, time to first examination, D-dimer and troponin I. Estimates are reported as odds ratios (ORs) and 95% confidence intervals (CIs). For secondary sensitivity analyses, we performed two parsimonious multivariable models for hemodynamic compromise and clinical presentation separately, adjusted for age and sex. Predictors of time to completion of diagnostic workup (logarithmically transformed) were assessed using separate univariable linear regression. Coefficients and 95% CIs were exponentiated and reported as time ratios (e.g., 0.7 indicates a 30% shorter time to completion of diagnostic workup).

Survival was assessed using a univariable Cox proportional hazards to explore survival relative to type A acute aortic syndrome, age, sex, hemodynamic compromise, clinical presentation, D-dimer and troponin I.

## 3. Results

### 3.1. Patient Characteristics

The study population consisted predominantly of older male patients with arterial hypertension. Type A AD was the most frequently confirmed subtype. POCUS was performed in 62% of all patients. The median time to completion of diagnostic workup was 143 min, and in-hospital mortality was 14%. A detailed overview of population characteristics is presented in Table 1.

In the group that had been assessed with POCUS, time to completion of diagnostic workup was shorter than in the group without POCUS assessment, although the difference did not reach statistical significance (*p* = 0.069). POCUS was used more frequently in hemodynamically unstable patients (87.5% vs. 54.4%, *p* = 0.020) and in those with an unclear clinical presentation (40% vs. 7%, *p* = 0.003). Detailed characteristics of both groups are presented in Table 2. POCUS detected cardiac tamponade in 4 (8.9%) patients, aortic regurgitation in 17 (38%) patients, intimal flap in 24 (53%) patients and intimal flap in the descending aorta in 7 (16%) patients.

### 3.2. Predictors of POCUS Use

Logistic regression analysis showed that hemodynamic instability was associated with higher odds of POCUS use (*p* = 0.027), whereas a clear clinical presentation was associated with lower odds of POCUS use (*p* = 0.007). Odds ratios and confidence intervals for all examined variables are presented in Table 3. In separate exploratory models adjusted for age and sex, hemodynamic instability remained associated with higher odds (OR 5.94, 95% CI 1.21–29.08, *p* = 0.028) and clear clinical presentation with lower odds (OR 0.12, 95% CI 0.03–0.58, *p* = 0.008) of POCUS use.

### 3.3. Impact of POCUS Use on Time to Completion of Diagnostic Workup

In univariable analysis using a log-transformed linear model, we did not demonstrate a statistically significant effect of POCUS on time to completion of diagnostic workup (*p* = 0.097). Increasing age and urgent/yellow triage category (compared with immediate/red) triage were associated with longer time to completion of diagnostic workup, whereas the highest D-dimer quartile was associated with shorter time to completion of diagnostic workup. Sex, hemodynamic instability, clinical presentation, and troponin quartiles were not significantly associated with time to completion of diagnostic workup. Full univariable results are presented in Table 4 and exploratory mutivariable results (POCUS use adjusted for age, sex, and either clinical presentation or hemodynamic status) are presented in Table 5.

### 3.4. Survival Analysis

The univariable Cox proportional-hazards models (Table 6) showed that age was the only predictor of mortality. Each additional year of age was associated with an approximately 6% increase in the risk of death (*p* = 0.007).

## 4. Discussion

The characteristics of our study population resembled those reported in the International Registry of Acute Aortic Dissection (IRAD) [29], with the typical AAS patient being an older male with known arterial hypertension and type A AD the most common subtype. However, similarity to selected IRAD demographics does not by itself establish the representativeness of our small, single-center cohort, and comparisons with registry data should be interpreted with this limitation in mind.

POCUS was used in 62% of our cases, higher than the 52% reported a decade ago [24] and substantially higher than in IRAD, where echocardiography was the first imaging modality in 25% of cases. Time to completion of diagnostic workup in our study was shorter than that reported in IRAD (143 vs. 258 min). These cohorts are not directly comparable, because they differ in era (our data are from 2019 onward, whereas the earliest IRAD cases date to 1996), in healthcare systems, and likely in how diagnostic times were defined and measured. We therefore cannot infer that the shorter time in our cohort reflects the increasing adoption of POCUS in AAS evaluation; we offer this only as a hypothesis that requires prospective testing in comparable settings. The large, multicenter composition of IRAD (over 7300 cases) further limits direct comparability of findings [29].

POCUS was used significantly more often in hemodynamically unstable patients (87% vs. 54.4%, *p* = 0.020) and in those with an unclear clinical presentation (40% vs. 7%, *p* = 0.003). CTA was performed in nearly all patients (96%) to establish the definitive diagnosis, whereas POCUS served as a complementary bedside tool during the initial diagnostic assessment. The pattern of POCUS use suggests the possibility of confounding by indication: POCUS was not assigned randomly but rather used preferentially in patients who were clinically unstable or diagnostically challenging. These patients may inherently follow different diagnostic pathways and undergo different diagnostic workups compared with more stable patients with clearer presentations. Thus, the observed association between POCUS and diagnostic workup time may reflect, at least in part, the clinical circumstances prompting the use of POCUS rather than an effect of POCUS itself. In the primary analysis, POCUS was not associated with time to completion of diagnostic workup, whereas adjusted analyses yielded inconsistent estimates, further supporting cautious interpretation. Because residual confounding by indication cannot be excluded by the observational design of our study, our analysis cannot be interpreted as demonstrating a causal effect of POCUS use on diagnostic speed.

Urgent/yellow triage, compared with immediate/red triage, was associated with longer time to completion of the diagnostic workup, whereas higher D-dimer level (highest vs. lowest quartile) was associated with shorter workup time. We interpret these as potentially reflecting appropriate prioritization and a more recognizable clinical picture, but these are hypotheses rather than established mechanisms and require direct evaluation. Age at presentation emerged as the only predictor of mortality in our study cohort; given the relatively small study population, our findings can only suggest that age at presentation captures several underlying and unmeasured factors, such as frailty and comorbidities.

This study provides contemporary, real-world data on POCUS use in confirmed AAS from a single emergency unit over a five-year period, encompassing type A and type B AD as well as intramural hematoma. Our findings are consistent with previous studies identifying POCUS as a rapid, complementary tool in time-sensitive emergency settings [3,22]. Given the descriptive design of this study, these findings should be interpreted as associations that generate hypotheses rather than as evidence of causal effects. The absence of large-scale studies systematically evaluating determinants of POCUS use in confirmed AAS limits direct comparison and highlights a notable gap in the literature that warrants further investigation. Future multicenter prospective studies are needed to define the impact of POCUS on diagnostic pathways and clinical outcomes and to examine additional determinants of its use, including equipment availability, physician expertise, protocol standardization, and organizational factors such as CTA accessibility.

### Limitations

This study has several limitations. As a retrospective analysis, it relies on the completeness and accuracy of medical records. POCUS examinations may have been performed but not documented, particularly when findings were inconclusive or technically difficult, which could lead to underestimation of true POCUS use. In addition, POCUS is inherently operator-dependent: image acquisition, interpretation, and diagnostic reliability vary with physician experience and training. Because operator expertise was not consistently documented, variability in skill may have influenced both the performance and reporting of POCUS.

Although POCUS was documented as occurring during the initial emergency-unit evaluation before definitive diagnosis, exact timing relative to CTA was not consistently available. POCUS images were also not systematically archived or independently verified, so the documented findings could not be re-adjudicated.

Reconstruction of clinical presentation and AORTAs score was based on chart documentation, which differs in detail across physicians and shifts. Although two investigators independently reviewed each case, incomplete records may have introduced classification bias. Hemodynamic instability was defined using pragmatic criteria derived from available vital signs, which may not fully reflect clinical severity.

Case identification was based on ICD10 coding, and rapidly fatal or miscoded cases without definitive imaging may not have been captured. This may introduce selection bias and limit the cohort to patients who survived long enough to undergo CTA confirmation.

The sample size limited multivariable modelling; primary regressions were therefore univariable, with only parsimonious multivariable models performed as exploratory sensitivity analyses. An additional limitation is confounding by indication: POCUS was performed at the discretion of the treating physicians and was more frequently performed in unstable and diagnostically challenging presentations, which may in themselves affect the diagnostic workup and time. Hence, the study was also not designed to assess diagnostic accuracy or causal effects of POCUS, and study findings should be interpreted as real-world clinical exploration and hypothesis-generating rather than providing definite estimation of POCUS predictors and/or diagnostic utility. The relatively small sample size also limited statistical power to detect modest associations. Accordingly, non-significant findings, particularly the association between POCUS use and time to completion of diagnostic workup, should not be interpreted as demonstrating absence of an effect or equivalence between groups. The wide confidence intervals indicate substantial uncertainty in the magnitude of these associations.

Finally, this was a single center study conducted in a tertiary emergency medical unit with high POCUS expertise and CTA availability. Diagnostic pathways and POCUS utilization may differ in other settings, limiting generalizability.

## 5. Conclusions

In this single-center retrospective cohort of patients with confirmed AAS, POCUS was documented in 62% of cases and was more frequently used in patients with hemodynamic instability and unclear clinical presentations. POCUS use was not associated with time to completion of diagnostic workup in the primary analysis. These findings describe local practice patterns and should not be interpreted as evidence that POCUS improves diagnostic speed or clinical outcomes. Prospective multicenter studies with standardized examination protocols, operator documentation, and inclusion of patients evaluated for but ultimately not diagnosed with AAS are needed.

## Figures and Tables

**Table 1:** Patient characteristics.

Characteristic	n = 73 ^1^
Age (years)	70 (51, 77)
Sex (male)	52 (71%)
Arterial hypertension	38 (52%)
Marfan syndrome	3 (4.1%)
Triage category	
1 (red—immediate)	6 (8.2%)
2 (orange—very urgent)	45 (62%)
3 (yellow—urgent)	22 (30%)
Type of acute aortic syndrome	
Type A aortic dissection	48 (66%)
Type B aortic dissection	17 (23%)
Intramural hematoma	8 (11%)
POCUS performed	45 (62%)
CTA Performed	70 (96%)
D-dimer (μg/L)	4887 (2374; 21,272)
Unknown	12
Troponin (ng/L)	9 (5, 24)
Unknown	3
Beta blocker therapy	9 (12%)
Vasodilating therapy	25 (34%)
Time to exam (min)	0 (0, 51)
Time to completion of diagnostic workup (min)	143 (69, 429)
Hemodynamic instability	16 (22%)
Clear clinical presentation	53 (73%)
Surgery performed	44 (60%)
TEVAR performed	5 (6.9%)
Hospital death	10 (14%)

^1^ Median (IQR); n (%); Abbreviations: POCUS, point-of-care ultrasound; CTA, computed tomographic angiography; TEVAR, thoracic endovascular aortic repair.

**Table 2:** Characteristics according to POCUS use.

Characteristic	POCUS Not Performed, n = 28 ^1^	POCUS Performed, n = 45 ^1^	*p*-Value ^2^
Age (years)	67 (50, 75)	71 (59, 78)	0.6
Sex (male)	22 (79%)	30 (67%)	0.3
Arterial hypertension	15 (54%)	23 (51%)	>0.9
Marfan syndrome	3 (11%)	0 (0%)	0.053
Triage category			0.5
1 (red—immediate)	1 (3.6%)	5 (11%)	
2 (orange—very urgent)	17 (61%)	28 (62%)	
3 (yellow—urgent)	10 (36%)	12 (27%)	
Type of acute aortic syndrome			0.2
type A aortic dissection	15 (54%)	33 (73%)	
type B aortic dissection	8 (29%)	9 (20%)	
Intramural hematoma	5 (18%)	3 (6.7%)	
D-dimer (μg/L)	4887 (2939; 11,174)	4783 (2339; 23,497)	0.7
Troponin (ng/L)	10 (5, 34)	7 (5, 19)	0.6
Time to exam (min)	1 (0, 71)	0 (0, 36)	0.6
Time to completion of diagnostic workup (min)	213 (100, 537)	110 (57, 340)	0.069
Hemodynamic instability	2 (7.1%)	14 (31.1%)	0.020
Clear clinical presentation	26 (93%)	27 (60%)	0.003

^1^ Median (IQR); n (%). ^2^ Wilcoxon rank sum test; Fisher’s exact test. Abbreviations: POCUS, point-of-care ultrasound.

**Table 3:** Univariable association with POCUS use.

Characteristic	n	OR	95% CI	*p*-Value
Age (years)	73	1.01	0.98–1.04	0.561
Sex (male)	73	0.55	0.17–1.58	0.278
Triaged as immediate (red)	73			
1 (red—immediate)		Ref		
2 (orange—very urgent)		0.33	0.04–3.06	0.329
3 (yellow—urgent)		0.24	0.02–2.41	0.225
Hemodynamic instability	73	5.87	1.22–28.24	0.028
Clear clinical presentation	73	0.12	0.03–0.58	0.008
Time to exam	73	1.00	0.99–1.01	0.855
D-dimer quartile	61			
Q1 (<193 μg/L)		Ref		
Q2 (194–2374 μg/L)		0.69	0.16–2.88	0.606
Q3 (2375–4887 μg/L)		0.53	0.12–2.18	0.378
Q4 (>4887 μg/L)		1.65	0.36–8.14	0.521
Troponin quartile	70			
Q1 (<5 ng/L)		Ref		
Q2 (6–9 ng/L)		1.65	0.41–7.05	0.481
Q3 (10–23 ng/L)		0.91	0.23–3.56	0.890
Q4 (>23 ng/L)		0.72	0.18–2.75	0.626

Abbreviations: OR, odds ratio estimates; CI, confidence interval; Q, quartile.

**Table 4:** Univariable associations with time to completion of diagnostic workup.

Characteristic	n	Time Ratio	95% CI	*p*-Value
POCUS performed	73	0.65	0.39–1.09	0.097
Age (years)	73	1.02	1.00–1.04	0.018
Sex (male)	73	1.30	0.74–2.28	0.358
Triaged as immediate (red)	73			
1 (red—immediate)		Ref		
2 (orange—very urgent)		2.23	0.96–5.22	0.063
3 (yellow—urgent)		5.43	2.21–13.4	<0.001
Hemodynamic instability	73	0.76	0.41–1.40	0.368
Clear clinical presentation	73	0.72	0.41–1.27	0.245
D-dimer quartile	61			
Q1 (<193 μg/L)		Ref		
Q2 (194–2374 μg/L)		1.02	0.49–2.13	0.963
Q3 (2375–4887 μg/L)		0.66	0.31–1.38	0.260
Q4 (>4887 μg/L)		0.44	0.21–0.93	0.031
Troponin quartile	70			
Q1 (<5 ng/L)		Ref		
Q2 (6–9 ng/L)		1.36	0.66–2.80	0.405
Q3 (10–23 ng/L)		1.76	0.84–3.68	0.131
Q4 (>23 ng/L)		1.18	0.57–2.47	0.654

Abbreviations: CI, confidence interval.

**Table 5:** Sensitivity analysis: adjusted associations with time to completion of diagnostic workup.

Characteristic	Model 1: Clinical Presentation			Model 2: Hemodynamic Status		
	Time Ratio	95% CI	*p*-Value	Time Ratio	95% CI	*p*-Value
POCUS performed	0.54	0.32–0.91	0.022	0.69	0.41–1.16	0.158
Age (years)	1.02	1.01–1.04	0.006	1.03	1.01–1.04	0.003
Sex (male)	1.62	0.94–2.78	0.084	1.56	0.89–2.72	0.117
Clear clinical presentation	0.57	0.33–1.02	0.056			
Hemodynamic instability				0.76	0.41–1.40	0.375
R^2^	0.197			0.162		
Adjusted R^2^	0.149			0.113		
AIC	215			218		
BIC	228			231		
Model df	4.00			4.00		
Residual df	68.0			68.0		
N	73			73		

Abbreviations: CI, confidence interval.

**Table 6:** Univariable association with mortality.

Characteristic	n	HR	95% CI	*p*-Value
Type A AD	73	0.83	0.32–2.14	0.695
Age (years)	73	1.06	1.02–1.11	0.007
Sex (male)	73	1.06	0.38–2.97	0.914
Hemodynamic instability	73	1.52	0.54–4.26	0.430
Clear clinical presentation	73	0.93	0.33–2.61	0.883
D-dimer quartile	61			
Q1 (<193 μg/L)				
Q2 (194–2374 μg/L)		2.72	0.53–14.1	0.232
Q3 (2375–4887 μg/L)		3.16	0.64–15.7	0.158
Q4 (>4887 μg/L)		2.35	0.43–12.8	0.324
Troponin quartile	70			
Q1 (<5 ng/L)				
Q2 (6–9 ng/L)		1.00	0.20–4.96	0.999
Q3 (10–23 ng/L)		1.68	0.40–7.01	0.480
Q4 (>23 ng/L)		1.86	0.45–7.80	0.394

Abbreviations: HR, hazard ratio; CI, confidence interval.

## Data Availability

The original contributions presented in this study are included in the article. Further inquiries can be directed at the corresponding author.

## Abbreviations

The following abbreviations are used in this manuscript:

AAS	Acute aortic syndrome
AD	Aortic dissection
ADD-RS	Aortic dissection detection risk score
AORTAs	AORTAs simplified score
CTA	Computed tomography angiography
ICD-10	International classification of diseases
IMH	Intramural hematoma
IQR	Interquartile range
IRAD	International registry of acute aortic dissection
POCUS	Point of care ultrasound
